# Snack Portion Sizes for Preschool Children Are Predicted by Caregiver Portion Size, Caregiver Feeding Practices and Children′s Eating Traits

**DOI:** 10.3390/nu11123020

**Published:** 2019-12-10

**Authors:** Sophie Reale, Rebecca M. Simpson, Colette Marr, Sharon A. Carstairs, Joanne E. Cecil, Marion M. Hetherington, Samantha J. Caton

**Affiliations:** 1 School of Health and Related Research (ScHARR), University of Sheffield, Sheffield S1 4DA, UK; s.reale@shu.ac.uk (S.R.); r.simpson@sheffield.ac.uk (R.M.S.); c.kearney@sheffield.ac.uk (C.M.); 2 Population and Behavioural Sciences, School of Medicine, University of St Andrews, St Andrews KY16 9TF, UK; sc295@st-andrews.ac.uk (S.A.C.); jc100@st-andrews.ac.uk (J.E.C.); 3 School of Psychology, University of Leeds, Leeds LS2 9JT, UK; m.hetherington@leeds.ac.uk

**Keywords:** portion size, preschool children, snacks, high energy dense, low energy dense, caregivers

## Abstract

Caregivers are mostly responsible for the foods young children consume; however, it is unknown how caregivers determine what portion sizes to serve. This study examined factors which predict smaller or larger than recommended snack portion sizes in an online survey. Caregivers of children aged 2 to 4 years were presented with 10 snack images, each photographed in six portion sizes. Caregivers (n = 659) selected the portion they would usually serve themselves and their child for an afternoon snack. Information on child eating traits, parental feeding practices and demographics were provided by caregivers. Most caregivers selected portions in line with recommended amounts for preschool children, demonstrating their ability to match portion sizes to their child′s energy requirements. However, 16% of caregivers selected smaller than recommended low energy-dense (LED, e.g., fruits and vegetables) snacks for their child which was associated with smaller caregiver′s own portion size, reduced child food liking and increased satiety responsiveness. In contrast, 28% of caregivers selected larger than recommended amounts of high energy-dense (HED, e.g., cookies, crisps) snacks for their child which were associated with larger caregiver′s own portion size, greater frequency of consumption, higher child body mass index (BMI), greater pressure to eat and lower child food liking. These findings suggest that most caregivers in this study select portions adjusted to suit their child′s age and stage of development. Future interventions could provide support to caregivers regarding the energy and nutrient density of foods given the relatively small portion sizes of LED and large portions of HED snacks offered to some children.

## 1. Introduction

Caregivers shape children′s food preferences, consumption and general diet quality through modelling behaviours and the type and quantity of food they make available within the household [1]. Modelling or observational learning is an important way children learn about food, and this has been demonstrated to shape children′s food preferences and eating practices [2]. In a recent systematic review, caregiver modelling of food consumption was associated with similar food consumption and food beliefs in children [3]. Caregivers act as the ‘gatekeepers’ of paediatric nutrition, determining the amount of food to be offered, and developing social norms for the child [4].

Children′s meal and snack consumption is influenced by environmental and behavioural factors, such as food availability [5], the maternal diet [6], individual differences in eating traits (e.g., satiety responsiveness) [7] and parental feeding practices (e.g., pressure to eat) [2]. Caregivers often use specific practices to influence food intake and reinforce the development of eating patterns that they deem appropriate [2,8]. Feeding practices fall into two main themes: controlling (e.g., restriction or pressure to eat) or noncontrolling (e.g., provide child autonomy) [9] and generally are child-centred as an adaptive response to children′s eating traits, food fussiness and specific food problems [10,11,12]. For example, satiety responsiveness, defined as the child′s ability to reduce food intake in response to internal satiety cues [13], has been inversely related to energy intake [14,15]. In contrast, some feeding practices, such as modeling [2,16], are successful in promoting healthy consumption [17] whereas others (e.g., pressure to eat) can reduce desire to eat and actual consumption of a target food [18].

Portion size mothers serve their children at an evening meal was found to strongly correlate to the portion size they serve themselves [19]. However, generally there is a paucity of research exploring the relationship between caregiver portion sizes and child portion sizes in the UK, for both meals and snacks. Snack foods feature significantly in the habitual diet and are reported to contribute around 21% of children′s total daily energy intake in the UK [20] and USA [21]. Frequent consumption of large portion sizes of HED foods has been associated with a larger body mass index (BMI) in children aged 1–5 years [22] and 5–9 years [23]. Given that larger portion sizes lead to greater immediate energy intakes [24] and sustained intake over a five days period without compensation in children aged 3–5 years [25]; a better understanding of the factors that predict snack portion size selection may be useful for developing public health interventions to address child snack intake in the context of healthful eating. Data from the UK suggest that snack foods are offered to children in adult or larger than recommended portion sizes. For example, a UK based survey involving 1000 parents reported that 61% of parents offered their toddlers large portion sizes of jelly sweets (candy), with 24% of parents offering portions of sweets that were the equivalent to three times the recommended weekly amount, in one serving [26]. Similarly, in Scotland, 29% of parents offered their infants aged 8–12 months high energy dense (HED >2.5 kcal/g as defined by [27]) snack foods at least once per day, and the frequency of HED snack offerings increased with higher levels of social and economic deprivation [28].

Portion size recommendations and daily eating plans have been proposed for preschool children in the UK [29,30] and USA [31], to support caregivers in providing a nutritionally balanced diet to children. In 2015, More and Emmett [32] proposed evidence-based appropriate portion size ranges for a variety of foods, and a practical, balanced food plan for preschool children by combining published data from two national surveys (National Diet and Nutrition Survey [33] and Avon Longitudinal Study of Parents and Children [34,35]). Foods were allocated into five food groups (1. Bread, rice potatoes; 2. Fruit and vegetables; 3. Milk, yoghurt and cheese; 4. Meat, fish, eggs, nuts and pulses; and 5. Foods high in fat and/or sugar) and two food groups (groups 2 and 5) were split further to provide flexibility in serving frequencies and to reflect snack foods. Despite these guidelines being evidence-based, these recommendations are not easily accessible to the general public, and manufacturers tend not to state portion sizes for children on their products [36].

A recent systematic review [37] revealed that parental portioning practices at meals are influenced by caregiver portion size, perceived child hunger, child body size and caregiver employment status. However, to date, little is known about the associations between children′s snack intake, portion sizes and parental feeding practices. Furthermore, the review by Kairey et al. [37] comprised only three studies from the UK, of which none focused primarily on snack foods [38,39,40].

It is not yet clear what determines the snack portion size that caregivers serve to preschool children in the UK. The aims of the current investigation were to compare adult-selected portion sizes for preschool children against the suggested amounts proposed by More and Emmett [32], and to explore what factors related to child feeding and eating behaviours predict portion size selection of HED and LED snack foods using an online survey.

## 2. Materials and Methods

### 2.1. Participants

Caregivers of children aged 2 to 4 years old were recruited from across the UK via university emailing lists, social media advertisements (e.g., Facebook, Twitter) and within toddler groups in Sheffield. To be eligible for participation, caregivers had to confirm on the online consent form that they were ≥18 years old, responsible for the food their child consumed in the home environment and neither themselves nor their child had a food allergy. The study was reviewed and approved by the School of Health and Related Research Ethics committee at the University of Sheffield (#011913).

### 2.2. Procedure

Participants were invited to take part in an online survey hosted on Qualtrics (Version January 2018; Provo, UT, USA). The survey was designed to be accessible from both mobile and desk-based devices (Figure S1). Participants were recruited via emailing lists from the University of Sheffield, social media advertisements (e.g., Facebook, Twitter) and within toddler groups in Sheffield between March and October 2017. One caregiver per child completed the survey and caregivers were instructed to complete the survey with their eldest child in mind if they had more than one child between the age of 2 to 4 years. Within the survey, caregivers were presented with images and measurements of the bowl and plate which featured throughout the survey; a valid method of estimating portion sizes [41]. Caregivers were then presented with a scenario to imagine; “*It is 2:30pm, your child/you had a sandwich 2.5 h ago for lunch and they/you are now hungry. Please select which snack you would provide*” and in a randomised order, images of 10 individual snack foods were then presented on screen. There were two sets of images, one related to adult portion sizes and one related to children′s portion sizes. Each snack food was presented in six portion sizes and caregivers were asked to select the portion size they would serve to their child and themselves for an afternoon snack (Figures S2–S11). The images for adults and children were presented to participants in a counterbalanced order to reduce any order effects. The imaginary scenario was provided to maintain consistency across participants and to control situational factors that have been found to influence portion size selections, such as child hunger and proximity to last/next eating occasion [42]. Caregivers completed measures of food liking and frequency of consumption for each snack food presented, for themselves and their child. Finally, caregivers provided demographic information and completed subscales from questionnaires related to parental feeding practices and children′s eating traits. On completion, caregivers were provided with the opportunity to be entered into a prize draw and/or express an interest in completing a second study. The survey took approximately 20 min to complete.

### 2.3. Materials and Measures

#### 2.3.1. Food Items

For the online survey, two items from each food group, as defined by More and Emmett [32], were selected to ensure inclusion of sweet and savoury, unit and amorphous, and high and low energy dense snacks (Low < 2.5 kcal/ g, High > 2.5 kcal/g, [27]). The selected snack items (Table 1 and Table 2) were identified as being familiar and regularly consumed by children [26,34,43] and adults [27,43].

#### 2.3.2. Calculation of Portion Sizes

Adult and child recommended portion sizes were derived from WHO recommendations [44] for fresh fruit and vegetables for both children (40 g) and adults (80 g). For commercially available foods, recommended amounts for children were based on the portion sizes outlined by More and Emmett [32], as seen in Table 1. For adults, portion size information was taken from manufacturers′ recommendations, as seen in Table 2. The remaining five portion sizes, three above and two below the recommended portion size, were calculated on a log scale, ensuring equal increments between each portion size, as research indicates that sensory systems respond in a logarithmic fashion to objects in the external world [45], see Table 1 and Table 2. This is referred to as Weber′s Law [46] which suggests that as the size of a stimulus increases the just noticeable difference gets larger, usually in proportion to the stimulus magnitude. Similarly with previous research, portion sizes ranged between 40% and 400% of the recommended amount [47].

#### 2.3.3. Display of Food

Each snack food item was removed from its original packaging, preweighed to the nearest gram and photographed in the centre of a plain white plate (23 cm diameter) or bowl (18.3 cm diameter) [48,49] (Figure S1). The dishware sizes used in this study are identical to the dishware sizes presented in portion size resources for carers of UK preschool children [50]. Dishware size was kept constant throughout the study.

Each photograph was taken in a specialist media suite, under constant lighting using a digital camera. A knife and fork were placed next to the plate/bowl to act as a size cue. The camera was mounted on a tripod at a 45-degree angle, 60 cm above and 60 cm horizontally away from the centre of the plate to ensure consistency between stimuli [51]. A place mat with marked regions for placement of the dinner plate/bowl, knife and fork was fixed to the table, ensuring optimal visibility [52]. The same camera angle and distance from each food item was used throughout to ensure the apparent sizes of all food items remained constant across the stimuli [53].

Each snack food was photographed in six portion sizes based on the log scale developed. The stimuli were presented in a vertical line from smallest to largest for all foods presented. This presentation was chosen as it is the most suitable display for survey completion on a mobile device, allowing participants to scroll through and select the stimuli.

#### 2.3.4. Caregiver Portion Size

Caregivers reported the size of each snack they would serve themselves by selecting the portion size image online that most closely resembled the usual amount they would serve themselves. Self-served caregiver portion size was then used as a predictor variable for the amount that the caregiver would serve their child.

#### 2.3.5. Food Liking and Hunger

Snack food liking and caregiver current hunger were assessed online using a 10-point Likert scale, with left to right anchors indicating ‘not at all’ on the left and ‘extremely’ on the right [54]. For example, caregivers reported snack food liking for themselves and their child “*Please rate how much your child likes the following food items from 0 (not at all) to 10 (extremely)*”. For food liking, an additional response was provided for food items that had not been consumed before. Liking and hunger were included as predictors in the model since parents have been reported to respond to their children′s food preferences and appetite [55].

#### 2.3.6. Frequency of Consumption

In the online survey, caregivers were required to indicate how often they and their child usually consume each snack item using the scale derived from a validated Food Frequency Questionnaire (FFQ) in children [56]. For each snack item, participants selected either “Never”, “once a month”, “once a fortnight”, “once a week”, “6 days a week” or “every day”.

#### 2.3.7. Parent and Child Characteristics

Several measures of children′s eating traits and parental feeding practices were included in the online survey: one scale from the Child Feeding Questionnaire (CFQ) [57] (Parental responsibility); three scales from the Comprehensive Feeding Practices Questionnaire (CFPQ) [58] (restriction for health, restriction for weight control and use of pressure); two scales from the Child Eating Behaviour Questionnaire (CEBQ) [13] (Food responsiveness and Satiety responsiveness) and the adapted six-item Child Food Neophobia scale for use in preschool children (CFNS) [59]. Caregivers also reported time spent watching television or playing video games in hours per week, as a proxy for caregiver and child sedentariness [60]. Demographic variables including, caregiver age (years); self-reported height (cm) and weight (kg) (converted to BMI kg/m^2^); relationship to child; educational attainment; employment status; ethnicity; income; deprivation score (based on postcode, [61]); child age (months), sex (n = 131 (Data on child sex was collected from 131 participants since this variable had been omitted in error from early data collection period. The online survey was amended and child sex was added as a variable)) and parental reported child height (cm) and weight (kg) (BMI z-score were calculated using the WHO anthropometric calculator http://www.who.int/childgrowth/software/en/) were also included as potential predictors.

### 2.4. Data Analysis

All quantitative analyses were carried out using STATA (StataIC 15 (64-bit)). Responses for the LED snacks were combined by calculating the mean of the four LED portion sizes selected. Similarly, responses for all HED snacks were combined by calculating the mean of the six HED portion sizes selected. Data are presented as mean (±SD), percentages, odds ratios and confidence intervals for LED and HED foods.

Chi squared tests were run to identify if there was an association between portion size selection and snack energy density averaged across LED and HED foods, for children and adults. Significance was established at *p* < 0.05.

A multinomial logistic regression with robust standard errors (clustered at the participant level) was conducted to identify predictors of amounts caregivers selected for their child according to whether these were larger or smaller than recommended amounts. Next, subgroup analyses were conducted based on snack energy density (LED and HED). All variables were input into the model and removed individually using the backward step elimination method [62]. The final model contained only the variables that significantly increased the odds of selecting a small or large portion size compared to the recommended portion size. The recommended portion size [32,44] was assigned as the comparative variable; therefore, results are presented as the odds of selecting larger or smaller portion sizes compared to the recommended portion size. Responses regarding snack items that had not previously been consumed were recorded as missing data and not included in the analysis [63].

As part of a sensitivity analysis, missing (at random) [64] survey responses (<10%) from 11 demographic variables (child age, child BMI z-score, child screen time, adult age, adult BMI, adult screen time, hunger, education, employment, income and deprivation), were imputed (n = 50) using the multiple imputation method in STATA. The *mi impute chained* function was used with *regress*, *ologit* and *mlogit* for continuous, ordered categorical and unordered categorical variables respectively. Data was pooled using Rubin′s rules [65] and the parameter standard errors combined using the *mi estimate* command. This method was repeated for each subgroup analysis and the Wald statistic was used to identify which predictor to remove at each stage. The findings of the sensitivity analysis (data not shown) corresponded with the initial analysis; therefore, findings from the initial analysis are presented.

## 3. Results

### 3.1. Participant Characteristics

A total of 930 caregivers consented to participate in the online survey, of which 232 (25%) dropped out part way through survey completion and 698 (75%) completed the online survey in its entirety. Seventeen participants (2%) were removed due to outliers and 22 participants (3%) were removed due to missing (at random) data, resulting in a total of 659 caregivers (611 mothers, 37 fathers, four aunts, three foster carers, two grandmothers and two undeclared) of preschool-aged children with a mean age of 34.2 ± 4.7 years. Most caregivers had completed high school education (95% ≥A-level or equivalents), were employed (82%), white British (87%) from England (99%), and on average 39% were classified as being overweight (M = 25.3 ± 5.4 kg/m^2^). Three participants were from Scotland and two were from Wales. According to the index of multiple deprivation, caregivers were from diverse socioeconomic backgrounds; 39% residing in areas of the UK that fall below the median decile for deprivation [61] with 26% of caregivers earning below the average household income for 2017 [66]. Children had a mean age of 35.9 ± 9.2 months and, on average, had a healthy body weight (BMI centile 57.9 ± 32.0, z score 0.3 ± 1.3). Of those who reported the sex of their child (n = 131), this was female in 53% of the cases, as seen in Table 3.

### 3.2. Portion Size Selection; Smaller than, in Line or Larger than Recommendations

Caregivers selected portion sizes of HED and LED snacks for themselves and their child smaller, larger and in line with the suggested amounts [32], as seen in Table 4. Chi squared revealed a significant association between child portion size selection and snack energy density, with more (41%) caregivers selecting LED snacks for their children close to suggested amounts for preschool children compared to HED snacks (26%) (x^2^(2) = 621.79, *p* < 0.001), as seen in Table 4. Similarly, the study revealed a significant association between adult portion size selection and snack energy density, x^2^(2) = 31.67, *p* < 0.001. However, in contrast to child portion size selection, more caregivers (42%) selected HED snacks for themselves closer to recommendations for adults compared to LED snacks (36%), as seen in Table 3.

### 3.3. Predictors of Child Portion Size

Table 5 and Table 6 show the results of the multinomial logistic regression analyses for LED and HED snack foods, respectively. Each row provides statistics associated with the variables that predict smaller than recommended portion size selection (LED = adult portion size, child liking and child satiety responsiveness; HED = adult portion size, child liking, child frequency of consumption and monitoring) and larger than recommended portion size selection for preschool children (LED = adult portion size, adult food liking, child food liking, child satiety responsiveness and pressure to eat; HED = adult portion size, child food liking, child frequency of consumption, child BMI z-score and parental pressure to eat).

#### 3.3.1. LED Snacks

Caregivers who selected LED snacks in line (OR = 2.60, 95% CI = 1.98 − 3.41, *p* < 0.001) or larger (OR = 13.45, 95% CI = 9.90 − 18.28, *p* < 0.001) than recommended portion sizes for themselves were 2.6 and 13.5 times more likely to select a large portion size for their child, as seen in Table 4. Furthermore, the odds of caregivers serving large portion sizes of LED snacks were increased by 17% and 14% respectively, with increases in child food liking (OR = 1.17, 95% CI = 1.11–1.23, *p* < 0.001) and pressure to eat (OR = 1.14, 95% CI = 1.02–1.26, *p* = 0.02). In contrast, the odds of caregivers serving large portion sizes of LED snacks were reduced by 13% and 19% with increases in adult liking (OR = 0.87, 95% CI = 0.84−0.92, *p* < 0.001) and child satiety responsiveness (OR = 0.81, 95% CI = 0.68−0.95, *p* = 0.01), as seen in Table 5.

#### 3.3.2. HED Snacks

Caregivers who selected HED snacks in line (OR = 2.38, 95% CI = 1.80–3.15, *p* < 0.001) with recommended or larger (OR = 5.58, 95% CI = 4.12–7.53, *p* < 0.001) portion sizes for themselves were 2.4 and 5.6 times more likely to select a larger than recommended portion size for their child, respectively, as seen in Table 5. The odds of caregivers serving larger than recommended portion sizes of HED snacks were increased by 22% with increases in child frequency of consumption (OR = 1.22, 95% CI = 1.16–1.28, *p* < 0.001), 3% with increased child BMI z-score (OR = 1.03, 95% CI = 1.00–1.06, *p* = 0.02) and 11% with increased pressure to eat (OR = 1.11, 95% CI = 1.01–1.21, *p* = 0.03). In contrast, the odds of caregivers serving smaller than recommended portion sizes of HED snacks were increased by 13% with increases in child frequency of consumption (OR = 1.13, 95% CI = 1.08–1.18, *p* < 0.001) and by 22% with monitoring (OR = 1.22, 95% CI = 1.10–1.38, *p* < 0.001). Increases in child food liking reduced the odds of selecting both smaller and larger than recommended portion sizes (OR for smaller = 0.86, CI = 0.83−0.90, *p* < 0.001; OR for larger = 0.95; CI = 0.91–0.99, *p* < 0.05), as seen in Table 6).

## 4. Discussion

The aims of the current investigation were to compare adult-selected portion sizes for preschool children against the suggested amounts proposed by More and Emmett [32] and to explore what factors related to child feeding and eating behaviours predict portion size selection of HED and LED snack foods using an online survey.

Most caregivers identify portion sizes of LED snacks in line with and larger than the suggested amounts for children and themselves. Similarly, the majority of caregivers were likely to serve themselves and their child HED snacks in portion sizes in line with, or smaller than, the suggested amount. However, a proportion of adults were selecting larger than suggested and smaller than suggested portion sizes of HED and LED snacks, respectively. The results of the multinomial logistic regression demonstrate that caregiver portion size, reported child liking, pressure to eat, child satiety responsiveness, caregiver monitoring, child frequency of consumption and child BMI z-score were significant predictors of LED and HED child snack portion size selection.

Overall, caregivers adapted portion sizes for the age and size of their child which were similar to the portion sizes suggested by More and Emmett [32], thus demonstrating their ability to downsize portion sizes to match their preschool children′s energy requirements. However, 31% and 16% of caregivers selected smaller than recommended portion sizes of LED snacks for themselves and their child, respectively. Almost a third of caregivers (data not shown) selected portion sizes of HED snacks up to four times the recommended amount for adults and children [32] in one serving, for themselves and their child. These findings are consistent with previous UK survey results [26] demonstrating that some preschool children are being served large portions sizes of HED snack foods, in many cases the equivalent to three times the weekly recommended amount. Similarly, adults are typically consuming larger amounts of HED snacks than on pack portion size suggestions [67]. Frequent exposure to large portion sizes is associated with a sustained increase in energy intake over a five day period in children aged 3–5 years [25] and over 11 days in adults [68] suggesting that exposure to large portion sizes might contribute towards excess energy intake and, ultimately to a positive energy balance.

Caregiver portion size was positively associated with child snack portion size. This suggests that for all of the included snacks, caregivers tend to judge appropriate portion sizes for their child, in line with their own self-selected portion size. This finding extends previous US-based research that identified a positive association between adult and child portion size at an evening meal [19]. Positive associations between child and adult portion size may be due to social norms [69], food availability [5,70], parental food liking [19] or parental hunger [54]. For example, maternal feelings of hunger influence maternal perceptions of their child′s hunger and thus the amount of food mothers served their children at a buffet style meal, regardless of their child′s actual hunger levels [54].

In the present study, increased child food liking was associated with reduced odds of both smaller and larger than recommended HED portion size selections meaning that caregivers are more likely to provide their child with a recommended portion size when the child likes the snack item. Qualitative research suggests that caregivers consider their child′s food preferences and requests when preparing meals by responding to their child′s individual differences [38,55,71]. Some caregivers consider that adjustments to portion size should be made according to nutritional content, such that HED foods should be limited [38]. Therefore, caregivers may be reluctant to offer large portion sizes of HED snacks, despite them being highly liked, as they choose to prioritize their child′s health and nutritional intake over their child′s food preferences. However, this remains to be further investigated, especially in a more varied sample

Related to child liking is frequency of consumption, since foods that are well-liked by children are generally offered more frequently [71]. In the present study, frequency of consumption was not a significant predictor of LED snack portion size. However, increased frequency of consumption predicted increased odds of selecting both smaller and larger than recommended portion sizes of HED snack foods. For example, caregivers who report that their child frequently consumes HED snacks might offer this snack in smaller than recommended portions sizes, possibly in an attempt to monitor their child′s snack intake. Conversely, other caregivers are demonstrating more permissive feeding practices and offering frequent and large portion sizes of HED snacks, a strategy which has previously described as a means of controlling preschool children′s behavior [26].

Child BMI z-score also predicted larger than recommended portion sizes of HED snacks, so the higher the child BMI z-score the more likely their caregiver was to select a larger than recommended portion size of HED snacks. These findings support previous literature demonstrating a positive association between portion size of HED snacks and BMI [22,72,73]. It is possible that caregivers serve children with a higher BMI z-score larger snack food portion sizes to meet their greater energy needs; however, this warrants further investigation as the direction of causality remains unknown.

Monitoring food intake, a controlling feeding practice, was a significant predictor of smaller than recommended portion sizes of HED snacks. Parental monitoring has been associated with reduced purchases of HED foods [74] and increased offerings of fruits and vegetables [75]. Monitoring intake might be a successful strategy to limit overconsumption of HED snacks if not offered frequently. In contrast, pressure to eat was associated with increased odds of selecting large portion sizes of HED and LED snacks, suggesting that caregivers may offer large food portion sizes to promote consumption. Pressure to eat is often demonstrated in circumstances where caregivers want their child to eat a certain type of food (usually fruits and vegetables) or a larger quantity of food [76]. However, the literature consistently demonstrates counter-productive effects of pressure to eat, whereby children tend to consume less of the target food rather than more [77,78].

Satiety responsiveness, a trait associated with responsiveness to feelings of fullness and good internal self-regulation [79], was associated with increased odds of selecting smaller than recommended portion sizes of LED snacks, as well as reduced odds of selecting larger than recommended portion sizes of LED snacks. This suggests that parents may be applying a child-centred strategy to portion size for children scoring high on satiety responsiveness. Caregivers learn from past feeding experiences and respond to their child′s appetite to provide portion sizes in line with the quantity they believe their child will accept and consume at meal times [38,55,71,80]. For example, in a qualitative study exploring the aspirations and challenges of feeding preschool children, mothers stated that they determine mealtime portions by honoring and valuing their child′s food preferences and trusting their child to stop consuming a meal when full [55]. Similar portioning practices may be apparent when LED snacks are on offer as caregivers may be conscious of food waste from an environmental or financial cost perspective [81]. 

### Strengths/Limitations

The present study primarily represents maternal portioning practices due to the greater number of female respondents (94%), exemplifying the dominant role female caregivers play in shaping young children′s dietary intake [1], or alternatively, the increased likelihood of female research participation. Moreover, the sample reflects a white British (87%) and highly educated (72% university degrees) group of mothers. Future work would benefit from recruiting a more heterogeneous sample in order to more fully understand predictors of portion sizes across different groups. The chosen research design allowed for multiple snack foods to be assessed within a single test session, online. The ease of participation increased statistical power meaning the findings could be used to understand the relative importance of variables that influence snack portion size selection.

Despite the advantages, screen-based measures may misinform actual portion size selection and consumption, when a mismatch occurs between expected and actual food properties [82]. For example, snack food items were removed from their packaging and provided on a plate/bowl, thus observed as 2D objects without exposure to sensory characteristics such as taste, smell and visual cues including packaging which may influence snack food selection and consumption [83,84]. Furthermore, an even number of snack food images were presented to reduce a central tendency effect; however, images were displayed in order of size, from smallest to largest, which may have influenced portion size selection towards the middle points of the range. Nevertheless, there was sufficient variability to test differences in portion sizes by a number of child and parent-related predictors. In the current study, manufacturer′s portion sizes were used as the recommended portion sizes for adults. However, Lewis et al. [85] demonstrated a lack of consistency between portion sizes that are communicated to the public. Future work would benefit from incorporating more than one publicly recommended portion size into the analysis to further validate the current findings.

The present study examined the snack portion size that caregivers select for their young children without addressing possible second servings or snack variety. Research suggests that some mothers choose to offer a small portion size in the first instance, knowing that their child will ask, and thus receive, more [38]. Therefore, it is possible that the portion sizes selected in this online study may not reflect the entire quantity children receive or consume at one snack occasion. This may be improved in future studies by adapting the scenario description (i.e., telling respondents to consider the full amount of food given). Moreover, whilst BMI was accounted for, the energy needs of participants were not accounted for in the analysis. Similarly, from the current design, we could not establish how the current findings relate to habitual energy intake. These aspects warrant further investigation.

Data on child sex was only collected from 131 participants due to this variable being missing from the early data collection period. Of this smaller sample, 53% of the participant population were female which is a good representation of the UK population, of which 51% are female [86]. Moreover, a sensitivity analysis was carried out only on those data where sex data was available. Sex was not demonstrated to be a significant predictor in our study population.

## 5. Conclusions

Overall, in the current sample of UK-based caregivers, most adapted portion size to the age and size of their child. However, 16% and 31% of the sample selected smaller than recommended portions sizes of LED snacks for their child and themselves, respectively, and 28% selected larger than recommended portion sizes of HED snack foods for themselves and their children. Significant predictors of child portion size selection included: caregiver′s own portion size selection, child characteristics including reported child liking, child satiety responsiveness, child BMI z-score, child frequency of consumption and parental feeding practices such as pressure to eat and caregiver monitoring. These findings suggest that caregivers in this sample used a child-centred approach to portion control, with most offering portions adjusted to child age and size. In the future, interventions could focus on encouraging caregivers to offer more and larger portions of LED nutrient dense foods, such as fruits and vegetables, as snacks, while limiting portion sizes of HED snack foods.

## Figures and Tables

**Table 1:** Weight and energy of each snack item presented in the online survey for children aged 2–4 years.

		Weight (g) of Portion Size (Energy in Kcal)					
Food Item	Energy Density (kcal/g)	Portion Size 1	Portion Size 2	Portion Size 3 *	Portion Size 4	Portion Size 5	Portion Size 6
Cucumber ^a^	0.16	16 (3)	25 (4)	40 (6)	64 (10)	101 (16)	160 (26)
Carrot ^a^	0.42	16 (7)	25 (11)	40 (17)	64 (27)	101 (42)	160 (67)
Gala Apple ^a^	0.53	16 (8)	25 (13)	40 (21)	64 (34)	101 (54)	160 (85)
White Grapes ^a^	0.66	16 (11)	25 (17)	40 (26)	64 (42)	101 (67)	160 (106)
White Toast ^b^ (Hovis ©)	2.56	11 (28)	17 (44)	27 (69)	43 (110)	68 (177)	108 (276)
Swiss Roll ^b^(Strawberry and cream, Tesco own brand)	3.64	9 (33)	14 (51)	23 (83)	36 (131)	57 (205)	90 (328)
Cereal ^b^ (Cornflakes, Kellogg′s ™, ^®^, ©)	3.78	7 (26)	11 (42)	18 (68)	29 (110)	45 (170)	72 (272)
Chocolate coated cookie ^b^ (Digestives, McVities ^®^)	4.95	6 (30)	9 (45)	15 (74)	24 (119)	38 (188)	60 (297)
Salted potato chips ^b^ (Walkers ©)	5.26	4 (21)	6 (32)	10 (53)	16 (84)	25 (132)	40 (210)
Mini milk chocolate buttons ^b^ (Cadbury′s, Mondelez ©)	5.35	3 (16)	5 (27)	8 (43)	13 (70)	20 (107)	32 (171)

^a^ LED, ^b^ HED [27], * recommended amounts as proposed by More and Emmett [32] and World Health Organisation [44].

**Table 2:** Weight and energy of each snack item presented in the online survey for adults.

		Weight (g) of Portion Size (Energy in kcal)					
Food Item	Energy Density (kcal/g)	Portion Size 1	Portion Size 2	Portion Size 3 *	Portion Size 4	Portion Size 5	Portion Size 6
Cucumber ^a^	0.16	32 (5)	50 (8)	80 (13)	127 (20)	202 (32)	320 (51)
Carrot ^a^	0.42	32 (13)	50 (21)	80 (34)	127 (53)	202 (85)	320 (134)
Gala Apple ^a^	0.53	32 (17)	50 (27)	80 (42)	127 (67)	202 (107)	320 (170)
White Grapes ^a^	0.66	32 (21)	50 (33)	80 (53)	127 (84)	202 (133)	320 (211)
White Toast^b^ (Hovis ©) ^b^	2.56	16 (41)	25 (64)	40 (102)	64 (164)	101 (259)	160 (410)
Swiss Roll ^b^(Strawberry and cream, Tesco own brand)	3.64	13 (47)	20 (73)	32 (116)	51 (186)	81 (295)	128 (466)
Cereal ^b^ (Cornflakes, Kellogg′s ™, ^®^, ©)	3.78	12 (45)	19 (72)	30 (113)	48 (181)	76 (287)	120 (454)
Chocolate coated cookie ^b^ (Digestives, McVities ^®^)	4.95	14 (69)	22 (109)	35 (173)	56 (277)	88 (436)	140 (693)
Salted potato chips ^b^ (Walkers ©)	5.26	10 (53)	16 (84)	25 (132)	40 (210)	63 (331)	100 (526)
Mini milk chocolate buttons ^b^ (Cadbury′s, Mondelez ©)	5.35	11 (59)	18 (96)	28 (150)	45 (241)	71 (380)	112 (599)

^a^ LED, ^b^ HED [27], * recommended amounts from the World Health Organisation [44] and manufacturer′s information.

**Table 3:** Participant demographics.

Child	Demographic Information
Gender	47% male
Age (months)	35.87 ± 9.16
**Caregiver**	
Age (years)	34.18 ± 4.72
BMI (kg·m^2^)	26.30 ± 5.40
Relationship to child	93% Mothers
	6% Fathers
	<1% Grandmothers
	<1% Aunts
	<1% Foster Carer
Ethnicity	87% White British
	1% White Irish
	8% White other
	<1% Black African
	<1% Asian Indian
	<1% Asian Pakistani
	<1% Asian other
	<1% White and Black Caribbean
	<1% Chinese
	2% Other
Highest Education	5% CSE, GCSE or O-Level (primary education)
	12% GNVQ, BTEC or A-Level (secondary education)
	11% Higher national certificate or diploma (further education)
	8% Undergraduate university degree
	34% Postgraduate university degree
Employment Status	15% Maternity leave
	3% Unemployed
	15% Stay at home to look after the children
Income	2% £ 0–10,000
	8% £ 10–20,000
	16% £ 20–30,000
	21% £ 30–40,000
	53% £ 40,000+

**Table 4:** Percentage of caregivers who selected low energy dense (LED; e.g., fruits and vegetables) and high energy dense (HED; e.g., cookies and crisps) snack portion sizes in line with, smaller or larger than recommended amounts for preschool children and adult.

Portion Size Selected	LED (n = 659)		HED (n = 659)	
	Child Portion Size	Adult Portion Size	Child Portion Size	Adult Portion Size
Smaller than recommended	105 (16%)	205 (31%)	303 (46%)	198 (30%)
In line with recommended	271 (41%)	237 (36%)	171 (26%)	277 (42%)
Larger than recommended	283 (43%)	217 (33%)	185 (28%)	184 (28%)

**Table 5:** Variables that predict portion size selection of LED snack foods and odds ratio for the likelihood of selecting a smaller or larger portion size compared with recommendations (n = 2620 ^a^).

**Smaller than Recommended**		
**Predictors**	**Odds Ratio**	**95% CI**
Adult PS in line (vs. small)	0.20	0.15–0.27
Adult PS above (vs. small)	0.15	0.09–0.25
Child Liking ^b^	0.83	0.79–0.88
Satiety Responsiveness	1.23	1.00–1.51
**Larger than recommended**		
Adult PS in line (vs. small)	2.60	1.98–3.41
Adult PS above (vs. small)	13.45	9.90–18.28
Adult Liking ^b^	0.87	0.84–0.92
Child Liking ^b^	1.17	1.11–1.23
Satiety Responsiveness	0.81	0.68–0.95
Pressure to eat	1.14	1.02–1.26

^a^ Based on 659 participants and four individual LED snack food items (carrot, cucumber, gala apple and white grapes), 16 responses missing at random or removed due to a snack food item not being consumed before, ^b^ Based on a 10 point Likert scale, Reference category = recommended snack portion size.

**Table 6:** Variables that predict portion size selection of HED snack foods and odds ratio for the likelihood of selecting a smaller or larger portion size compared with recommendations (n = 3399 ^a^).

**Smaller than Recommended**		
**Predictors**	**Odds RATIO**	**95% CI**
Adult PS in line (vs. small)	0.51	0.42–0.63
Adult PS above (vs. small)	0.31	0.24–0.39
Child Liking ^b^	0.86	0.83–0.90
Child frequency of consumption^c^	1.13	1.08–1.18
Monitor	1.22	1.10–1.38
Larger than recommended		
Adult PS in line (vs. small)	2.38	1.80–3.15
Adult PS above (vs. small)	5.58	4.12–7.53
Child Liking ^b^	0.95	0.91–0.99
Child frequency of consumption ^c^	1.22	1.16–1.28
Child BMI z-score	1.03	1.00–1.06
Pressure to eat	1.11	1.01–1.21

^a^ Based on 659 participants and six individual HED snack food items (cereal, chocolate coated cookie, mini milk chocolate buttons, salted potato chips, swiss roll and white toast), 555 responses missing at random or removed due to a snack food item not being consumed before, ^b^ Based on a 10 point Likert scale, ^c^ Mean frequency of consumption per week based on the Food Frequency Questionnaire (FFQ) scale [56], Reference category = recommended snack portion size.

## Supplementary Materials

The following are available online at https://www.mdpi.com/2072-6643/11/12/3020/s1, Figure S1: Online mobile display for the survey employed in this study, Figure S2: Portion size stimuli of cucumber, Figure S3: Portion size stimuli of carrot, Figure S4: Portion size stimuli of gala apples, Figure S5 Portion size stimuli of white grapes, Figure S6: Portion size stimuli of white toast (unbuttered), Figure S7: Portion size stimuli of dry breakfast cereal, Figure S8: Portion size stimuli of swiss roll, Figure S9: Portion size stimuli of chocolate coated cookie, Figure S10: Portion size stimuli of salted potato chips, Figure S11: Portion size stimuli of milk chocolate buttons.
