# Supplementary material for: Snack Portion Sizes for Preschool Children Are Predicted by Caregiver Portion Size, Caregiver Feeding Practices and Children′s Eating Traits
Source: Nutrients. 2019 Dec 10;11(12):3020. doi: 10.3390/nu11123020 (PMC6950396; doi:10.3390/nu11123020)
Supplement: Supplementary file 1 [file nutrients-11-03020-s001.pdf]

**Supplementary Materials:**

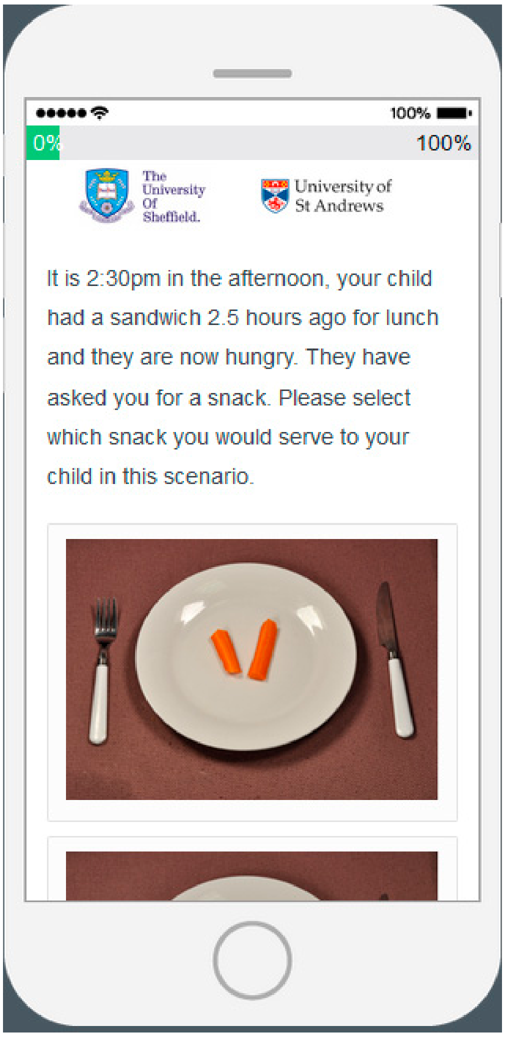


Figure S1: Online mobile display for the survey employed in this study.

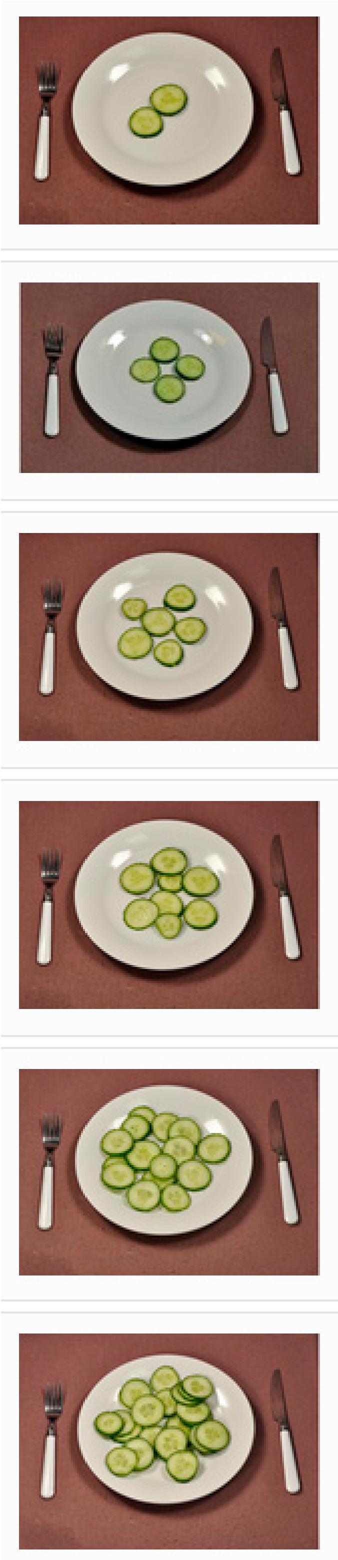

Figure S2: Portion size stimuli of cucumber

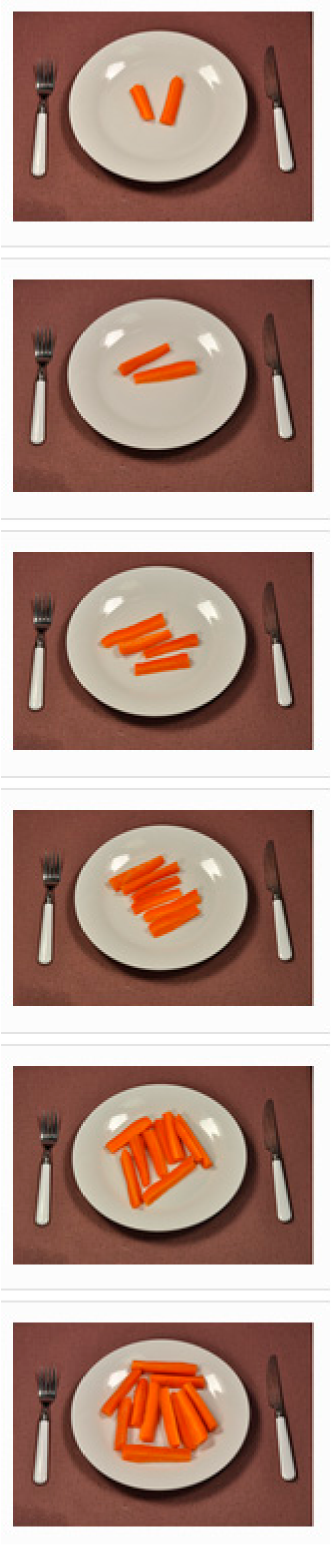

Figure S3: Portion size stimuli of carrot

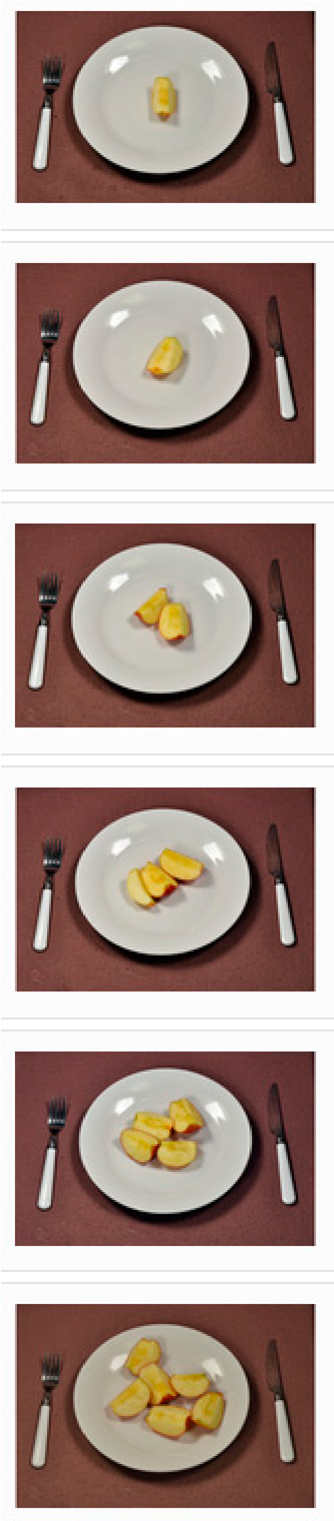

Figure S4: Portion size stimuli of gala apples

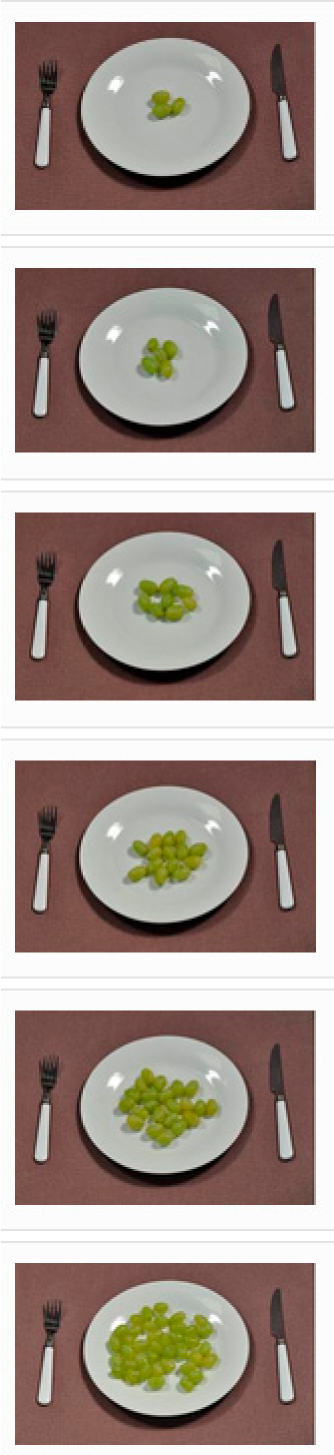

Figure S5: Portion size stimuli of white grapes

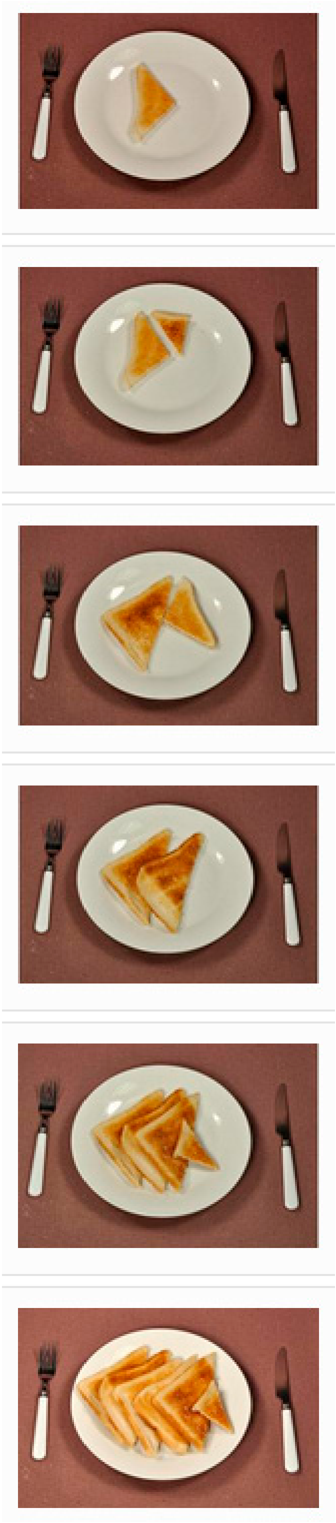

Figure S6: Portion size stimuli of white toast (unbuttered)

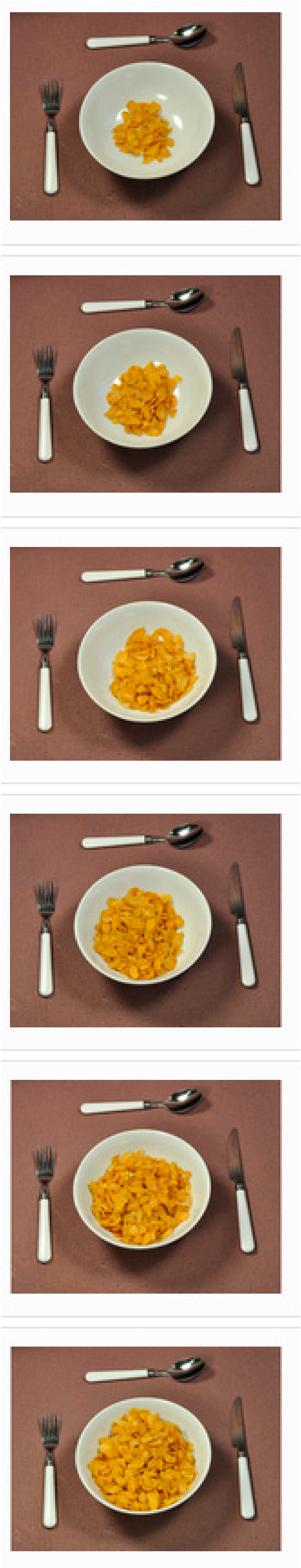

Figure S7: Portion size stimuli of dry breakfast cereal

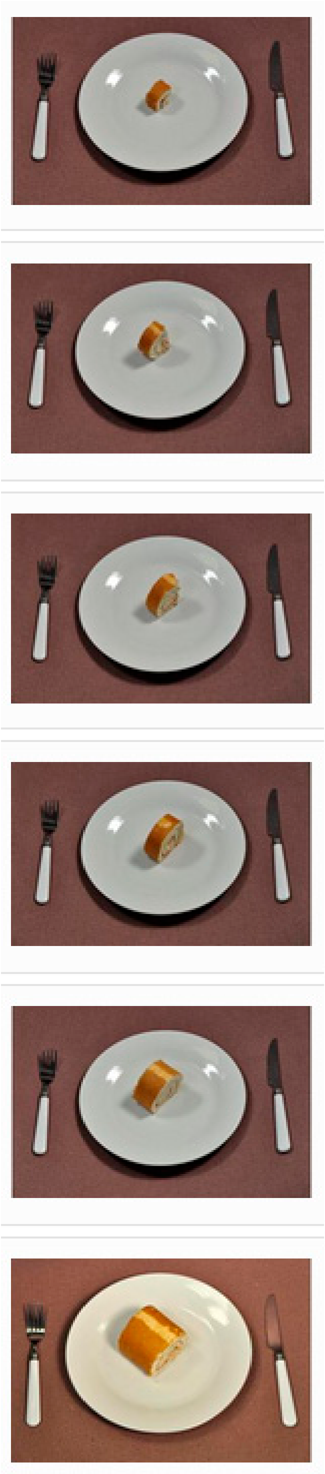

Figure S8: Portion size stimuli of swiss roll

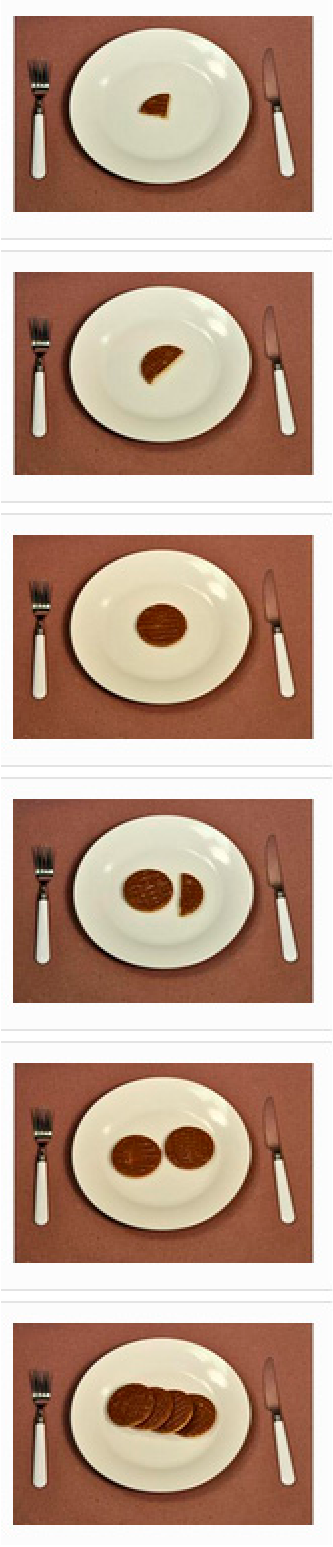

Figure S9: Portion size stimuli of chocolate coated cookie

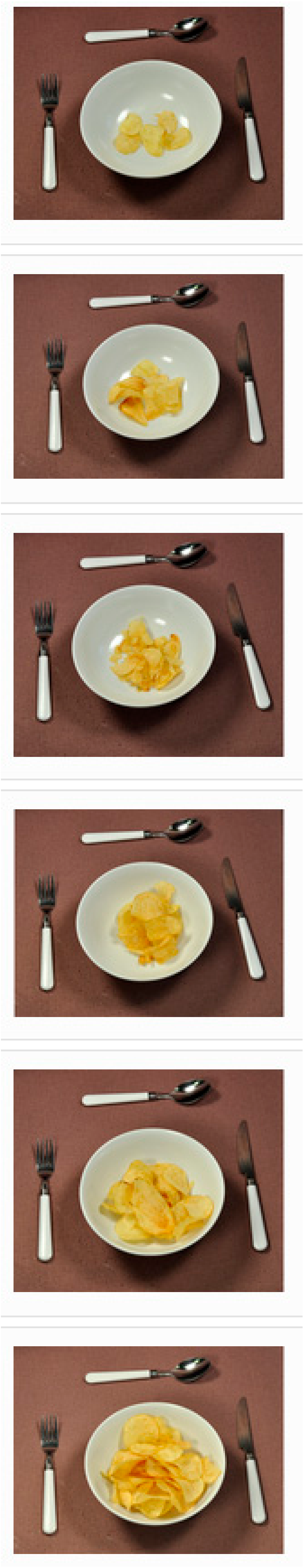

Figure S10: Portion size stimuli of salted potato chips

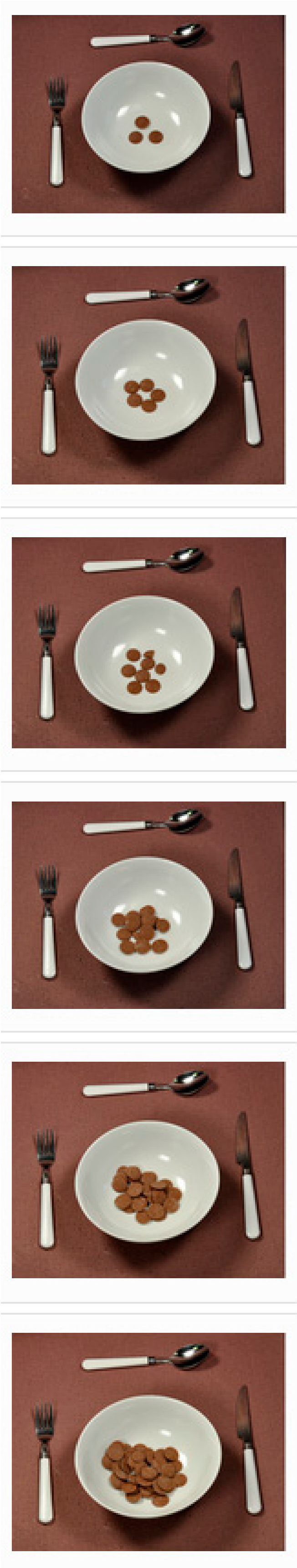

Figure S11: Portion size stimuli of mini milk chocolate buttons
